# Conservation and management of the culture of bears

**DOI:** 10.1002/ece3.8840

**Published:** 2022-04-19

**Authors:** Christopher Servheen, Kerry A. Gunther

**Affiliations:** ^1^ 307078 W.A. Franke College of Forestry and Conservation University of Montana Missoula Montana USA; ^2^ Bear Management Office Yellowstone Center for Resources Yellowstone National Park Wyoming USA

**Keywords:** behavior, conservation, culture, grizzly bear, management, *Ursus arctos horribilis*, Yellowstone National Park

## Abstract

Culture is widely accepted as an important social factor present across a wide range of species. Bears have a culture as defined as behavioral traditions inherited through social learning usually from mothers to offspring. Successful bear cultures can enhance fitness and resource exploitation benefits. In contrast, some bear cultures related to response to humans and human‐related foods can be maladaptive and result in reduced fitness and direct mortality. In environments with minimal human influence most bear culture has evolved over generations to be beneficial and well adapted to enhance fitness. However, most bears across the world do not live in areas with minimal human influence and in these areas, bear culture is often changed by bear interactions with humans, usually to the detriment of bear survival. We highlight the importance of identifying unique bear cultural traits that allow efficient use of local resources and the value of careful management to preserve these adaptive cultural behaviors. It is also important to select against maladaptive cultural behaviors that are usually related to humans in order to reduce human–bear conflicts and high bear mortality. We use examples from Yellowstone National Park to demonstrate how long‐term management to reduce maladaptive bear cultures related to humans has resulted in healthy bear populations and a low level of human–bear conflict in spite of a high number of Yellowstone National Park visitors in close association with bears.

## Graphical abstract

Bears have a culture as defined as behavioral traditions inherited through social learning usually from mothers to offspring. Successful bear cultures can enhance fitness and resource exploitation benefits. We highlight the importance of identifying unique bear cultural traits that allow efficient use of local resources and the value of careful management to preserve these adaptive cultural behaviors.

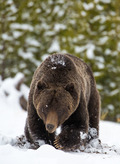

## 1 INTRODUCTION

Culture is the inheritance of an array of behavioral traditions through social learning from others (Whitten, 2021). There is a rich literature on the culture of inherited behaviors across a wide range of taxa emphasizing its evolutionary significance and adaptive importance. This literature includes: song dialects in white‐crowned sparrows (*Zonotrichia leucophrys*) (Marler & Tamura, 1964), foraging sites in birds (Slagsvold & Wiebe, 2011), whale songs (Noad et al., 2000), bottlenose dolphin (*Tursiops* spp.) foraging traditions (Mann & Sargeant, 2003), humpback whale (*Megaptera novaeangliae*) feeding methods (Allen et al., 2013), capuchin monkey (*Cebus* spp.) tool use (Ottoni et al., 2005), sperm whale (*Physeter macrocephalus*) dialects (Weilgart & Whitehead, 1997), killer whale (*Orcinus orca*) ecotype evolution (Foote et al., 2016), killer whale diets, foraging strategies, and social conventions (Boran & Heimlich, 1999; Rendell & Whitehead, 2001), ungulate migration (Jesmer et al., 2018), and social transmission of prey avoidance between predators (Thorogood et al., 2018).

## 2 CULTURE IN BEARS

Culture exists in bears (*Ursidae*) and can include foraging locations and techniques, selection of different foods, habitat preferences, seasonal movement patterns and use areas, behavioral responses including avoidance or attraction to various factors including humans, human activities such as hunting, human‐related food sources, and perhaps den locations and den types.

In humans, cultures are complex and diverse and persist within and between areas and the groups of people in those areas. Human culturally related behaviors and customs are reinforced if they confer resource exploitation benefits, financial benefits, and/or increased social acceptance (Richardson & Christiansen, 2013). Similarly, behaviors inherited due to bear culture persist because they can confer resource exploitation benefits. In both humans and bears, beneficial socially learned cultural behaviors persist, adapt, and evolve over time.

Culture in bears is displayed by behaviors transmitted through social learning usually from mothers to offspring because bears have such a long period when offspring and mothers stay together. Human food‐conditioned bear foraging behavior is usually transmitted within family groups, through social learning, from mother bears to cubs, and from their grown female offspring to their cubs and future cubs (Cole, 1976; Gilbert, 1999; Mazur & Seher, 2008). Cubs learn preferred foods by watching their mother and sharing their mother's food during the 1.5–3.5 years spent under her care (Gilbert, 1999; Meagher & Fowler, 1989). In many ways, a bear's behavior and its food and habitat preferences mirror the behaviors and preferences of its mother.

Culture in bears in a natural environment free from human influences is shown by what can be termed “natural” behaviors such as food habits, seasonal movements, and habitat use. In such pristine environments, free of human influences, the culture of bears reflects long‐term evolutionary adaptation to resource exploitation and responses to factors that result in increased survival and reproductive success. In such pristine environments, cultural behaviors that confer increased survival and fitness are passed on from generation to generation.

The evolution of animals like bears is shaped by the combination of genetic inheritance and cultural inheritance and both are constantly subjected to evolutionary pressures over time (Whitten, 2021). Behavior in all animals is a combination of genetic inheritance that reflects the long‐term evolutionary responses of past behaviors on fitness and to the behavioral flexibility that allows animals to adapt their behavior based on recent experience (Breed & Sanchez, 2010). Morehouse et al. (2016) in an elegant approach tested the genetic versus learned origins of conflict behavior in grizzly bears (*Ursus arctos horribilis*) in southwest Alberta. They genotyped parents and their offspring and compared the frequency of problem (conflicts with humans or depredation on livestock) and non‐problem offspring from problem and non‐problem parents. They found no evidence that offspring from problem fathers were more likely to be involved in conflicts. In contrast, they documented that offspring of problem mothers were likely to also be conflict bears, whereas offspring of non‐problem mothers were not likely to be problem bears. Since young bears spend so much time with their mothers, social mother–offspring learning is the primary cause of problem behaviors related to human conflicts in young bears. Morehouse et al.'s (2016) results support nurture (mother–offspring teaching) as the primary source of problem behaviors in young bears rather than nature (inherited behaviors due to genetics).

Successful cultures in both humans and animals are those that allow beneficial exploitation of resources and increased survival of young. This may result in diverse cultures in bears, even those living in the same general areas and different foraging, or movement or habitat use behaviors within the same environments. For example, we know of areas in the Rocky Mountains south of Canada where certain groups of grizzly bears move to site‐specific alpine talus slopes in mid‐summer to feed on aestivating army cut worm moths (*Euxoa auxilliaris*) (Chapman et al., 1955; French et al., 1994; White et al., 1998). Only a small portion of the grizzly bear population in these areas use these cutworm moths even though they are a rich and nutritious food source. The home ranges of bears using and not using these cutworm moths often overlap. Presumably, use of the remote, specific alpine sites where such moths occur is a culturally transmitted behavior from mothers to offspring. We are not aware of any documentation of the mitochondrial DNA relationships and diversity in those bears that use alpine areas for cutworm moths, but it may be that the females using these remote sites are related as mothers/daughters/grandmothers. Related groups of female bears using such sites may possess reduced mitochondrial DNA diversity like what has been found in macaques (*Macaca* spp.) (Melnick & Hoelzer, 1992) and some whales with matrilineal social systems (Whitehead et al., 2017). Such related groups of bears would share the culture of alpine moth use learned from their mothers and passed on to their offspring. A similar presumably culturally inherited behavior occurs in Yellowstone National Park (YNP) where only a small proportion of the grizzly bear population feeds on spawning cutthroat trout (*Oncorhynchus clarkia*) (Haroldson et al., 2005). The limited numbers of female bears using trout may also be related and thus have reduced mitochondrial DNA diversity.

Bears are intelligent and adaptive to the range of stimuli they experience over their lifetime, so a bear's behavior and what it will pass on to its offspring is not just what it has been taught by its mother (Breck et al., 2008). The lifetime experiences of each bear are a combination of behaviors molded by positive outcomes, such as food acquisition, and negative outcomes, such as a fear of more dominant bears (Elfstrom et al., 2012; Nevin & Gilbert, 2005), avoidance of humans (Nellemann et al., 2007), or changes in nocturnal behavior when near humans (MacHutchon et al., 1998; Schwartz et al., 2010). When female bears have cubs, they will teach their cubs a combination of what they learned from their mothers and siblings (sibling pairs after weening) combined with new behaviors from their own life experiences (Gilbert, 1999; Madison, 2008; Meagher & Fowler, 1989), although Breck et al. (2008) reported that the transmission of human food preference from mothers to cubs was not universal. This combination of culturally inherited and learned behaviors is their culture that they pass on to their offspring. In the human‐dominated environment where most of the world's bears live, they constantly encounter humans and human‐associated activities that are powerful determinants of their survival and eventually to the survival of their offspring (Servheen et al., 2021).

Given that bears are intelligent and adaptable (Gilbert, 1999; Mazur & Seher, 2008), they can and do modify their behaviors and response to stimuli based on experience with humans (Herrero et al., 2005; Nevin & Gilbert, 2005; Smith et al., 2005). Many of these responses to human‐related activities are the result of learned cultural responses transmitted from mothers to offspring (Cole, 1976; Gilbert, 1999; Hopkins, 2013; Mazur & Seher, 2008; Meagher & Fowler, 1989). Thus, we see bears whose avoidance response to human presence over time is reduced by habituation (Aumiller & Matt, 1994; Herrero et al., 2005; Jope, 1985; McCullough, 1982) or bears that have obtained human‐related foods either through intentional feeding or careless human behavior who become food conditioned and begin to seek human use areas for feeding opportunities (Herrero, 2002; Mazur & Seher, 2008; Meagher & Phillips, 1983). Such behaviors represent human‐influenced bear cultures, which are often productive for bears in the short term (Can et al., 2014; Craighead et al., 1995) but maladaptive in the long term (Gilbert, 1999; Mazur & Seher, 2008; Meagher & Fowler, 1989).

## 3 THE MANAGEMENT AND CONSERVATION OF BEAR CULTURE

In the human‐dominated landscapes that most wildlife including bears inhabit in the world today, culture begins with some level of mother–offspring learning and is then modified by experience with humans (Laland, 2004; Servheen et al., 2021). Human actions that impact bear learned behaviors and culture may include human presence or absence, hunting, roads and vehicle use, human facilities and developments, human‐related foods and garbage, and human behavior such as humans who might approach and/or feed them (Krofel et al., 2021).

We normally think of human influences on bears primarily as modifiers of survival due to increased direct morality and indirectly through habitat modification and habitat fragmentation. Because humans are potent modifiers of bear behavior and culture, we have a responsibility to manage our behaviors and activities to attempt to minimize corrupting bear culture. Such human corruption of bear culture usually results in lowered reproductive success and increased mortality (Northrup et al., 2012). Consideration of bear culture is important because few bears across the world today live in pristine environments free of human influences and corruption of bear cultures.

YNP is an example of an area whose objective is to maintain as many natural or “pristine” bear cultures and behaviors as possible. However, with increasing human visitation, there are multiple challenges to bear managers to avoid cultural corruption, such as bears becoming human food conditioned and damaging property or injuring people to obtain anthropogenic foods. Managers in YNP have implemented management actions to reduce cultural contamination of YNP bears by: (1) providing YNP visitors with information on how to hike, camp, recreate, and store anthropogenic bear attractants in a manner that reduces the chances of bear–human conflicts; (2) providing YNP visitors with bear‐resistant infrastructure (e.g., bear‐resistant garbage cans, food storage devices, etc.) so that food and garbage storage regulations are easy and convenient to comply with; (3) rigorously enforcing food and garbage storage regulations through campground food security patrols and backcountry campsite patrols; and (4) removing (killing or sending to zoos) rather than translocating bears that intentionally seek out and damage property or injure people while attempting to obtain anthropogenic foods (in benign incidents where bears inadvertently happen upon unsecured foods they are left to roam free on the landscape).

YNP managers attempt to break the chain of learned conflict behavior passed from mothers to offspring and adult female offspring to future offspring (Cole, 1976; Meagher & Fowler, 1989). Breaking the sequence of learned conflict behaviors is important so that conflict behavior, such as damaging property or injuring people to obtain anthropogenic foods, does not become a traditional or cultural behavior that persists across multiple generations of matriarchal linages in a large segment of the bear population (Mazur & Seher, 2008). Once a conflict bear has been removed (killed or placed permanently in captivity), the next bear to reoccupy that habitat, area, or general range may be an immigrating subadult that exhibits wild behaviors rather than human food‐conditioned conflict behaviors (Cole, 1976; Meagher & Fowler, 1989). If the next bear to occupy the area exhibits conflict behaviors, it is also removed. The removal of human food‐conditioned bears exhibiting conflict behaviors allows young bears that are not conditioned to human foods to recruit into and progressively replace conflict bears in the local population (Cole, 1976; Meagher & Fowler, 1989). By consistently implementing this strategy over the long term, a population of bears once dominated by conflict behaviors, such as YNP from the 1930s to 1960s (Cole, 1971, 1976; Meagher & Phillips, 1983; Schullery, 1992; Wondrak‐Biel, 2006), can be converted into and maintained as a population composed of individuals exhibiting primarily wild behaviors (Cole, 1976), such as YNP from 1980 to 2021 (Garshelis et al., 2017; Gunther, 1994; Meagher & Phillips, 1983). Occasional removal of food‐conditioned bears is still sometimes necessary, as bear innovators periodically reestablish conflict behaviors (Hopkins, 2013; Mazur & Seher, 2008).

A comparison of a 19‐year (1981–1999) period in YNP when relocation of conflict bears was common, to a 22‐year (2000–2012) period when relocation was rarely used, provides opportunity to compare the efficacy of each management practice. High numbers of human‐caused grizzly bear mortalities in the early 1970s (Craighead et al., 1974; Craighead et al., 1988) resulted in GYE grizzly bears being listed as a threatened species in 1975. Knight and Eberhardt (1984, 1985) developed a model which suggested that the population was still in decline in the early 1980s, and that stability might hinge on the survival of just one or two additional adult female bears. As a result, the survival of individual grizzly bears, especially adult females, became a management priority and was considered crucial to conservation of the species (Meagher & Fowler, 1989). From 1981 to 1999, use of relocation of human food‐conditioned bears as an alternative to removal (killing or sending to zoos) became a standard management practice in YNP. In the mid‐1990s, counts of adult female grizzly bears in YNP stabilized, suggesting the subpopulation of grizzly bears in YNP likely had reached ecological carrying capacity. Beginning in 2000, because the park population had stabilized and to foster a bear population that did not seek anthropogenic foods, YNP began removing rather than relocating most human food‐conditioned bears. From 1981 to 1999, when relocation of bears involved in conflicts was a common management practice, there were 51,571,106 visits to YNP. During this period, there were 2.4 conflicts per 1 million visits where grizzly bears damaged property or obtained anthropogenic foods, 1.1 relocations per 1 million visits where grizzlies involved in conflicts were captured and relocated away from conflict sites, and 0.2 removals per 1 million visits where human food‐conditioned grizzlies were removed from the population. From 2000 to 2021, when most food‐conditioned bears were removed and relocation of conflict bears was rarely used, there were 76,137,667 visits to YNP. During this period, there were 1.3 conflicts per 1 million visits, 0.04 relocations of human food‐conditioned bears per 1 million visits, and 0.07 removals of human food‐conditioned bears per 1 million visits. Comparing the two time periods suggests that deferring removal of conflict bears through relocation results in higher rates of both conflicts and management removals over time. Cubs raised by mothers exhibiting conflict behaviors often cause conflicts themselves after weaning (Mazur, 2015; Mazur & Seher, 2008). In addition, survival of relocated food‐conditioned bears was low. Of 29 individual food‐conditioned bears that were relocated 60 times from 1981 to 2021, 12 caused further conflicts and were eventually removed in management actions, 12 had unknown fates, 2 were killed in defense of life and property incidents outside of YNP, 1 was poached outside of YNP, 1 was killed by a black bear hunter outside of YNP, and 1 was struck and killed by a vehicle. None of the 17 bears with known fates survived and died of natural causes. Survival of relocated conflict black bears (*Ursus americanus*) was also low in Yosemite, Sequoia, and Kings Canyon National Parks (Hopkins & Kalinowski, 2013; Mazur, 2015).

The YNP management strategy has been highly successful at reducing grizzly bear–human conflicts and management removals of grizzly bears on national park lands where humans are temporary visitors and their activities are highly controlled (Garshelis et al., 2017; Gunther, 1994; Meagher & Phillips, 1983; White et al., 2017). For example, during the last decade (2010–2019), there were 37.8 million recreational visits to YNP. These visitors spent >7.2 million overnight stays in roadside campgrounds, >400,000 overnight stays in remote backcountry campsites, and an estimated 2.6 million person–recreation days hiking in backcountry bear habitat in YNP. Given the high level of human recreational activity in YNP during the last 10 years, grizzly bears undoubtedly had some opportunities to come into conflict with people. Despite intense efforts to prevent bears from obtaining human foods, on any given night there was likely a bear‐resistant dumpster with a broken latch, a few coolers left out overnight in roadside campgrounds, or food that was not properly stored in backcountry campsites. However, under YNP's strategy of aggressively removing human food‐conditioned bears and promoting occupation of habitat by bears that are not conditioned to human foods, few bears in YNP sought anthropogenic attractants or tested bear‐resistant infrastructure. From 2010 to 2019, there were only 29 ($\bar{x}$= 2.9 ± 1.9 SD/year) incidents in the park where grizzly bears obtained human foods or damaged property while attempting to access anthropogenic attractants. Of those 29 incidents, 7 were benign and involved bears consuming anthropogenic foods that were left out and not stored in a bear‐proof manner. In 22 incidents, bears aggressively sought human foods or garbage and damaged property in the process. In response to those 22 incidents, 3 ($\bar{x}$= 0.3 ± 0.5 SD/year) independent age grizzly bears were removed (2 killed and 1 sent to a zoo) in management actions. It is important to note that limiting lethal management removals of bears to sustainable rates while operating under YNP's aggressive bear management strategy requires significant investment of resources in conflict prevention.

Capturing bears that come into conflict with people and translocating them to areas more remote from human developments is a common practice among bear managers throughout North America (Craven et al., 1998; Miller & Ballard, 1982; Milligan et al., 2018, and others). Translocation of conflict bears may enhance recovery and range expansion of populations listed as sensitive, threatened, or endangered. Indeed, translocation likely played an integral role in population recovery and range expansion of grizzly bears in the Greater Yellowstone Ecosystem after the high mortality associated with closure of garbage dumps in the region. However, once a population of bears has recovered from sensitive, threatened, or endangered status, removal of bears involved in conflicts may be much more cost effective than repeated translocations of bears raised in a culture of conflict behaviors. In a recovered population, aggressive removal of conflict bears can promote occupation of available habitat by bears that do not seek human foods, thereby reducing conflicts and generating more tolerance of bears and more support for protection of bear habitat.

Some aspects of bear culture are reflective of natural behaviors that in some cases are unique and vulnerable to human disturbance. These culturally inherited behaviors are sometimes site and season specific and need to be carefully managed so that they can persist. A good example is the use of army cutworm moth sites by grizzly bears in high‐elevation alpine habitats. In years past, these moth sites were favored by bear hunters who could go to hunt these sites and shoot grizzlies when they were very vulnerable in the open alpine habitat (French et al., 1994). Grizzly bears at such sites are sensitive to any type of human presence and will flee such alpine areas when climbers or hikers pass through on the way to climbing mountain peaks (Nunlist, 2020; White, 1996). Concern about human disturbance of grizzly bears feeding on alpine insect concentrations prompted the Confederated Salish and Kootenai Tribes to close the areas around McDonald Peak in Montana's Mission Mountains from July 15 to September 30 each year to allow the bears use of these open alpine habitats with no human disturbance (Klaver et al., 1986). In some areas where bears concentrate to feed on spawning salmon, wildlife managers have implemented management to reduce human disturbance by area closures, regulations that direct proper human behavior at bear viewing areas, or strict oversight of visitors like that at McNeil River in Alaska (Aumiller & Matt, 1994). Other examples include seasonal recreational activity closures in areas of YNP where grizzlies concentrate to feed on ungulate carcasses in spring, prey on elk (*Cervus canadensis*) calves or cutthroat trout in late spring and early summer, and feed on the seeds of whitebark pine (*Pinus albicaulis*) in late summer and fall (Coleman et al., 2013; NPS, 1982).

In summary, bear culture passed from mothers to offspring is an important basis of bear behavior and adaptation to their environment. In pristine habitats free of human influence, bear culture represents maternal teaching of behaviors and responses that improve survival and long‐term fitness. In much of the world, however, bear culture is tainted by human influences that can produce behaviors and responses that are maladaptive to survival and fitness. Bear managers must often act to select against such maladaptive behaviors by selectively increasing mortality of those female bears who will pass on these maladaptive cultural behaviors to their offspring. Humans can modify or disturb valuable natural cultural behaviors. Careful management is necessary to conserve and enhance the natural cultural behaviors that still exist so they can continue to be passed down from generations of mother bears to their offspring.

## CONFLICT OF INTEREST

None declared.

## AUTHOR CONTRIBUTIONS

**Christopher Servheen:**Conceptualization (lead); Data curation (supporting); Formal analysis (supporting); Investigation (equal); Methodology (equal); Writing – original draft (lead); Writing – review & editing (equal). **Kerry A. Gunther:** Conceptualization (supporting); Data curation (lead); Formal analysis (lead); Investigation (equal); Methodology (equal); Writing – original draft (supporting); Writing – review & editing (equal).

## Data Availability

The data that support the findings of this study are available from the corresponding author upon reasonable request.
